# Impact of the Akwenda Intervention Program for cerebral palsy on caregiver‐perceived burden, stress, and psychosocial functioning: A cluster‐randomized trial in Uganda

**DOI:** 10.1111/dmcn.16368

**Published:** 2025-06-14

**Authors:** Elizabeth Asige, Gillian Saloojee, Godfrey Wanjala, Carin Andrews, Lukia H. Namaganda, Angelina Kakooza‐Mwesige, Katherine Albus, Diane L. Damiano, Hans Forssberg

**Affiliations:** ^1^ Department of Pediatrics and Child Health Makerere University Kampala Uganda; ^2^ CURIE Study Consortium Iganga‐Mayuge Health and Demographic Surveillance System Iganga Uganda; ^3^ Department of Physiotherapy, Faculty of Health Sciences University of the Witwatersrand Johannesburg South Africa; ^4^ Department of Women's and Children's Health Karolinska Institutet Stockholm Sweden; ^5^ Centre for Health and Sustainability, Department of Women's and Children's Health Uppsala University Uppsala Sweden; ^6^ Department of Epidemiology and Biostatistics Makerere University School of Public Health Kampala Uganda; ^7^ Rehabilitation Medicine Department, Clinical Center National Institutes of Health Bethesda MD USA; ^8^ Astrid Lindgren Children's Hospital Stockholm Sweden

## Abstract

**Aim:**

To evaluate the effectiveness of the Akwenda Intervention Program in reducing subjective burden, stress, and the psychosocial and family functioning of caregivers of children with cerebral palsy (CP) in rural Uganda, and its relationship to child outcomes.

**Method:**

This was a cluster‐randomized, controlled, single‐blind study of 100 caregivers of children and young people with CP (aged 2–23 years; 48 females) assigned to an intervention or control group. The Zimbabwe Caregiver Challenges Scale (ZCCS) was used to measure caregiver burden, while the Pediatric Quality of Life Inventory (PedsQL) Family Impact Module was used to assess caregiver psychosocial and family functioning. Customized questionnaires and video protocols evaluated caregiver knowledge and skills. Data were analysed using *t*‐tests and Pearson's rank correlation coefficients.

**Results:**

The ZCCS score decreased in the intervention group (*p* < 0.001) but increased in the control group, resulting in a large group difference (*p* < 0.001; Cohen's *d* = −3.2). The PedsQL total score (*p* < 0.001; Cohen's *d* = 0.56) and the health‐related quality of life (Cohen's *d* = 1.15) score and knowledge of CP increased more in the intervention group. Greater knowledge correlated with reduced ZCCS scores and improved health‐related quality of life. The improvement in caregiver outcomes correlated with improved child functioning, activity, and participation.

**Interpretation:**

The Akwenda Intervention Program effectively reduced caregiver burden and stress, and improved psychosocial functioning. Improved knowledge of CP, peer support, and stigma reduction contributed to these improvements.

## Graphical abstract

The Akwenda Intervention Program reduced caregiver burden and stress, and improved psychosocial functioning of caregivers of children with CP.

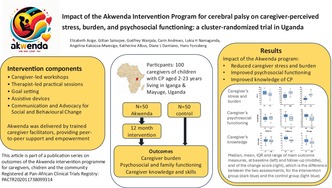

**This original article is commented by Newton on pages** 1115–1116 **of this issue.**

**Abbreviations**
HRQoL: health‐related quality of life
PedsQL: Pediatric Quality of Life Inventory
ZCCS: Zimbabwe Caregiver Challenges Scale

**What this paper adds**

- The Akwenda Intervention Program significantly improved the perceived biopsychosocial functioning of caregivers of children and young people with cerebral palsy (CP).
- The intervention reduced caregiver‐perceived burden of care and stress, probably leading to improved mental health.
- Caregiver CP‐related knowledge improved after the intervention, probably contributing to reduced burden and improved mental health.
- There was an interaction between improvements in caregiver outcomes and better child functioning, activity, and participation.

The caregivers of children with cerebral palsy (CP) endure higher levels of stress, anxiety, and depression, more physical symptoms, such as back pain and excessive fatigue, and have lower quality of life than the parents and caregivers of typically developing children.^1^, ^2^, ^3^ The mental and physical health of caregivers is influenced by caregiving demands, which in turn depend on the child's behaviour and physical condition. The social support provided by family, friends, neighbours, and public services, has a mediating influence and can promote better self‐perception and stress relief.^1^, ^4^, ^5^

The caregivers of children with disabilities in sub‐Saharan Africa are particularly vulnerable to stress, depression, and health issues because of several factors.^6^, ^7^, ^8^, ^9^ Limited resources and inadequate infrastructure result in insufficient societal support and health care services that could alleviate the burden on family caregivers.^10^ Often, the mother is solely responsible for the care of the child, lacking support from her spouse and other family members, thus reducing the time available for other household duties and income‐generating tasks.^6^, ^11^, ^12^ The caregivers of children with CP also face stigma and discrimination rooted in ignorance, superstitious beliefs, and negative attitudes towards disability.^6^, ^7^, ^8^, ^11^, ^13^, ^14^ Such societal attitudes can leave caregivers feeling insecure and isolated, making it difficult for them to access public services and support for fear of being judged or exposed.^12^ Another barrier to caregiver well‐being is the lack of understanding regarding their child's condition and care needs.^7^, ^15^, ^16^ Local beliefs frequently attribute disabilities to curses related to violations of social norms, supernatural forces, divine will, or other unexplainable phenomena.^11^, ^17^ These misconceptions may extend to health workers, leaving caregivers without accurate guidance or support. Consequently, many caregivers turn to traditional healers, herbalists, or religious leaders in an effort to improve their child's condition; these do not lead to meaningful improvement and contribute to caregivers losing hope and financial resources.^18^ Additionally, low formal education and literacy rates, particularly among women in rural areas, further hinder the ability of caregivers to access written information and resources that could assist in the care of their child.^11^

To meet the needs of children with CP in Uganda and their families, we developed the Akwenda Intervention Program; this is a multi‐component intervention programme with three goals: (1) to improve child functioning, activity, and participation; (2) to improve caregiver knowledge and skills, and improve mental health and quality of life; and (3) to reduce stigma and facilitate inclusion and participation. The Akwenda Intervention Program was developed by a team of international experts and South African and Ugandan academic and health professionals;^19^ it was inspired by a study from Ghana, which highlighted the impact of a caregiver training programme with participatory support groups on caregiver knowledge and empowerment.^12^, ^14^

In this study, we addressed the second goal of the Akwenda Intervention Program, that is, to improve caregiver knowledge and skills, and improve caregiver health and functioning. In previous publications, we reported outcomes at the child level, including improved social functioning, self‐care skills, and gross motor function, as well as improved participation in activities of daily living.^20^, ^21^ The hypothesis was that caregivers in the intervention group would demonstrate improved knowledge and skills, reduced burden and stress, and improved health and psychosocial functioning compared to a control group. We further explored associations between changes in caregiver knowledge or skills, and changes in burden, stress, self‐functioning, and family functioning, and between changes in these caregiver and child outcomes.^20^, ^21^

## METHOD

### Study setting and design

This article is the third in a series from a complex, real‐world, randomized, assessor‐blinded study of children and young people with CP from a low‐resource setting in Eastern Uganda. It was conducted over a 12‐month period from October 2021 to September 2022 at the Iganga‐Mayuge Health and Demographic Surveillance Site, which includes a population of approximately 90 000 residents across 65 villages, most of whom were engaged in subsistence farming and lived below or near the poverty line. The detailed methodology was published in a research protocol before the study's initiation, and in two reports on child outcomes.^19^, ^20^, ^21^

Ethical approval was obtained from the Uganda National Council for Science and Technology (SS‐5173 and HS 1979ES), and the Makerere University School of Public Health (HDREC 727) and School of Medicine and Ethics Committee (Mak‐SOMREC 2021–273). It was also registered with the Pan African Clinical Trials Registry (PACTR202011738099314). All caregivers provided written informed consent.

### Participants

This study included the caregivers of 100 children and young people aged 2 to 23 years (52 males) diagnosed with CP by a child neurologist according to the European CP surveillance criteria.^22^ Most (*n* = 65) were from a population‐based cohort identified in a three‐stage screening in 2015;^23^ an additional group of 35 caregivers of children aged 2 to 6 years was conveniently sampled from the same area. Caregivers and children living in the same and nearby villages were clustered together, resulting in two geographically defined clusters including 50 caregivers each. The rationale for this approach was to recruit families from villages in close proximity to the workshop venue for each of the four caregiver groups to facilitate transport and to minimize the risk of contamination of the communication and advocacy intervention. Participants for the two arms were recruited from 35 villages and 30 villages respectively, with each village contributing one to two participants. The two groups were stratified according to the child's age, sex, and Gross Motor Function Classification System (GMFCS) level.^24^ One group received the Akwenda Intervention Program, while the other served as a control group and was planned to receive the intervention the following year. A coin flip determined which group would receive the intervention first.

### The Akwenda Intervention Program

For a comprehensive overview of the Akwenda Intervention Program, see Appendix S1 or the research protocol.^19^ The programme consisted of five components: (1) caregiver‐led training workshops; (2) therapist‐led practical group sessions; (3) provision of assistive devices; (4) goal setting; and (5) communication and advocacy for social and behavioural change. The intervention group was divided into four subgroups based on geographical location. The intervention programme spanned 12 months and included seven caregiver‐led workshops, 14 therapist‐led practical sessions, two home visits, and four communication and advocacy for social and behavioural change sessions. Transport reimbursement and meals were provided to support participation. Attendance exceeded 90% for all sessions. The programme was coordinated by a physiotherapist and PhD student and implemented by four caregiver facilitators, three part‐time therapists, a social worker, and a community mobilizer.

Educating and training caregivers in a group setting using a participatory approach was a core component; this encouraged discussion and sharing of stories and experiences. Each group met twice a month: once in caregiver‐led workshops and once in therapist‐led sessions together with the children. Thus, caregivers learned how CP affected each child in a different way and how the principles and techniques covered in the workshop could be modified and adapted for each child. In these group sessions, problem‐solving in applying the principles taught was emphasized. The control group received the standard or usual care available for children with CP in the area. Children with seizures in both groups were provided with antiseizure medication when needed.

### Measurements

Outcome measures were collected by one team before group allocation and by another team at the follow‐up, who were blinded to group allocation. All questionnaires were translated to the local language (Lusoga) using forward and back translations by accredited language experts, and thereafter field‐tested on caregivers of children with CP to verify that the questions and terms used were well understood.

#### Caregiver knowledge

A custom‐designed questionnaire was developed based on the curriculum of the Malamulele Onward Carer‐2‐Carer Training Programme (Appendix S2).^25^ To establish the face validity of the questionnaire, five Ugandan caregivers and four Ugandan therapists were consulted. They were asked to: (1) evaluate whether, in their opinion, the items in the questionnaire accurately measured caregiver knowledge based on the content of the Carer‐2‐Carer training; and (2) assess each item for clarity (i.e. whether the question was clear and understandable), language appropriateness, and relevance. Based on their feedback and subsequent discussions, ambiguous questions were reworded and some of the language was simplified. The final version of the questionnaire included 36 statements, each with three possible responses: agree; disagree; or not sure. Correct responses were assigned a score of 1, while incorrect or unsure responses were assigned a score of 0, resulting in a total score range of 0 to 36.

#### Caregiver biopsychosocial and family functioning

The Pediatric Quality of Life Inventory (PedsQL) Family Impact Module measures the impact of chronic paediatric conditions on caregiver biopsychosocial and family functioning. It has high internal consistency (Cronbach's *ɑ* = 0.97 for the total scale; 0.96 for health‐related quality of life [HRQoL]; 0.90 for family functioning).^26^ The 36‐item questionnaire was administered to primary caregivers in their home, who responded according to a 5‐point Likert scale: never = 0; almost never = 1; sometimes = 2; often = 3; and almost always = 4. Item scores were then reversed and linearly transformed to a 0 to 100 scale, with higher scores indicating better functioning (www.pedsql.org/PedsQL‐Scoring.pdf). Items were subdivided into two main categories: caregiver HRQoL (20 items including self‐reported physical, emotional, social, and cognitive functioning) and family functioning (eight items including daily activities and family relationships). Communication (three items) and worry (five items) were included in the total score. Data were presented as mean scores for each individual, where the sum for each category was divided by the number of items answered.

#### Caregiver burden and stress

The care burden and stress of caregivers were assessed using the Zimbabwe Caregiver Challenges Scale (ZCCS),^27^ which was developed for caregivers of children with lifelong disabilities living in low‐resource settings. The ZCCS has demonstrated excellent internal consistency at both the subscale (α range: 0.77–0.84) and total scale (α = 0.93) levels and good reliability (interclass correlation coefficient = 0.88).^28^ The primary caregiver was interviewed in their home. The ZCCS contains 33 statements to which caregivers were asked to respond on a 5‐point Likert scale whether they ‘strongly agreed = 5’ to ‘strongly disagreed = 1’. Negatively worded statements were reverse‐coded before the analysis. Possible total scores ranged from 33 to 165; the higher the score, the higher the perceived burden of care. Subscale scores included: physical and economic burden (nine items); concerns for the child (14 items); family relations (five items); and community participation (three items).

#### Caregiver skills

We developed a custom‐made protocol to evaluate caregiver performance in feeding and dressing their child in the home setting (Appendix S3). A 3‐minute video of each activity was filmed on tablets or smartphones by the interviewer. These were later scored independently by two assessors blinded to group and time, with discrepancies between assessors resolved by discussions with a third trained assessor. Five behaviours in each video were scored according to a 4‐point Likert scale with a total range of 0 to 15. Before the study, assessors scored and discussed 10 practice videos, achieving high interrater reliability (>0.80).

#### Baseline characteristics and child outcomes

Sociodemographic information and clinical data, including anthropometric measurements and GMFCS^24^ and Manual Ability Classification System levels,^29^ were collected before group allocation. Both used a 5‐point ordinal scale from I (independent) to V (requires assistance in all activities).

Caregiver outcomes were correlated with the child outcomes reported previously,^20^, ^21^ including the 66‐item Gross Motor Function Measure,^30^ the Ugandan version of the Pediatric Evaluation of Disability Inventory,^31^ Picture My Participation,^32^ and with the age of the child and their GMFCS level.

### Statistical analysis

Data were analysed using SPSS v29.01.0 (IBM Corp., Armonk, NY, USA). Pearson's *χ* ^2^ and Fisher's exact tests were used (*p* < 0.05) to assess group differences across categorical variables describing participant baseline characteristics. Independent *t*‐tests were used to compare change scores across groups, while paired *t*‐tests were used to compare scores across time points within groups (*p* < 0.05, two‐tailed test). Pearson's rank correlation coefficients (*r*) were used to evaluate relationships across caregiver measures, and between caregiver and child outcomes. Spearman's rank correlation coefficients (*ρ*) were used to correlate child age and GMFCS level with caregiver outcomes.

A statistical power analysis based on PedsQL data from a previous intervention study in Ghana, involving caregivers of children with CP,^14^ was conducted before the study to ensure that the sample size was sufficient to detect differences between groups.^19^

## RESULTS

### Baseline demographics and functional levels

Ninety‐four participants completed the study; 48 in the intervention group and 46 in the control group (see the flow chart in Figure S1). Two participants in the intervention group withdrew, three children died, and one withdrew in the control group. The sociodemographic information of the primary caregivers is presented in Table 1 together with the basic characteristics of the children and young people with CP. The groups were similar in all sociodemographic and baseline characteristics (*p* > 0.05).

**TABLE 1:** Baseline demographics and clinical characteristics of caregivers and their children with cerebral palsy before randomization.

		All, *n* = 94 (%)	Intervention, *n* = 48 (%)	Control, *n* = 46 (%)	χ^2^	*p*
Primary caregiver						
Relationship to child	Mother	50 (53)	28 (58)	22 (48)	4.432	0.618
	Grandmother	25 (27)	10 (21)	15 (33)		
	Father	13 (14)	7 (15)	6 (13)		
	Other	6 (6)	3 (6)	3 (6)		
Occupation	Subsistence farmer	48 (51)	24 (50)	24 (52)	5.375	0.146
	Petty trade	33 (35)	18 (38)	15 (33)		
	Formal employment	4 (4)	3 (6)	1 (2)		
	Other	9 (10)	3 (6)	6 (13)		
Marital status	Married	64 (68)	33 (69)	31 (67)	0.802	0.670
	Separated	20 (21)	11 (23)	9 (20)		
	Widowed	10 (11)	4 (8)	6 (13)		
Educational level	Unknown	10 (11)	7 (14)	3 (7)	9.424	0.224
	None	5 (5)	1 (2)	4 (9)		
	Primary	53 (56)	24 (50)	29 (63)		
	Secondary	18 (19)	10 (21)	8 (17)		
	Tertiary/university	8 (9)	6 (13)	2 (4)		
Monthly income in Ugandan shillings^a^	< 100 000	55 (59)	29 (60)	26 (57)	7.736	0.102
	100 000–200 000	26 (28)	14 (29)	12 (26)		
	200 000–500 000	6 (6)	5 (10)	1 (2)		
	> 500 000	1 (1)	0 (0)	1 (2)		
	Unknown	6 (6)	0 (0)	6 (13)		
Age in years	26–45	43 (46)	21 (44)	22 (48)	3.308	0.191
	46–65	40 (42)	22 (46)	18 (39)		
	66–85	11 (12)	5 (10)	6 (13)		
Residence area	Semi‐urban	27 (29)	13 (27)	14 (30)	0.129	0.720
	Rural	67 (71)	35 (73)	32 (70)		
Child						
Age (years)	2–5	17 (18)	10 (21)	7 (15)	0.605	0.739
	6–12	48 (51)	23 (48)	25 (54)		
	13–23	29 (31)	15 (31)	14 (30)		
Sex	Female	42 (45)	19 (40)	23 (50)	1.031	0.310
	Male	52 (55)	29 (60)	23 (50)		
GMFCS level	I	25 (27)	16 (33)	9 (20)	6.608	0.158
	II	20 (21)	10 (21)	10 (22)		
	III	9 (10)	3 (6)	6 (13)		
	IV	21 (22)	8 (17)	13 (28)		
	V	19 (20)	11 (23)	8 (17)		
MACS level	I	22 (23)	15 (31)	7 (15)	4.490	0.344
	II	30 (32)	14 (29)	16 (35)		
	III	11 (12)	5 (11)	6 (13)		
	IV	12 (13)	4 (8)	8 (17)		
	V	19 (20)	10 (21)	9 (20)		

*Note*: Table 1 includes only those participating in both the baseline and follow‐up assessments. The table has been modified from a previous report on child outcomes from the same project.^20^ The Pearson's χ^2^ test was used to compare group differences at a statistical significance of *p* < 0.05.

Abbreviations: GMFCS, Gross Motor Function Classification System; MACS, Manual Ability Classification System.

^a^ Monthly household income: US$1 = 3500 Ugandan shillings. Poverty line = 85 170 Ugandan shillings per person per month. The Fisher's exact test revealed no statistically significant association between monthly income and group assignment (odds ratio = 0.244; *p* = 0.362).

### Knowledge about CP

**FIGURE 1:**
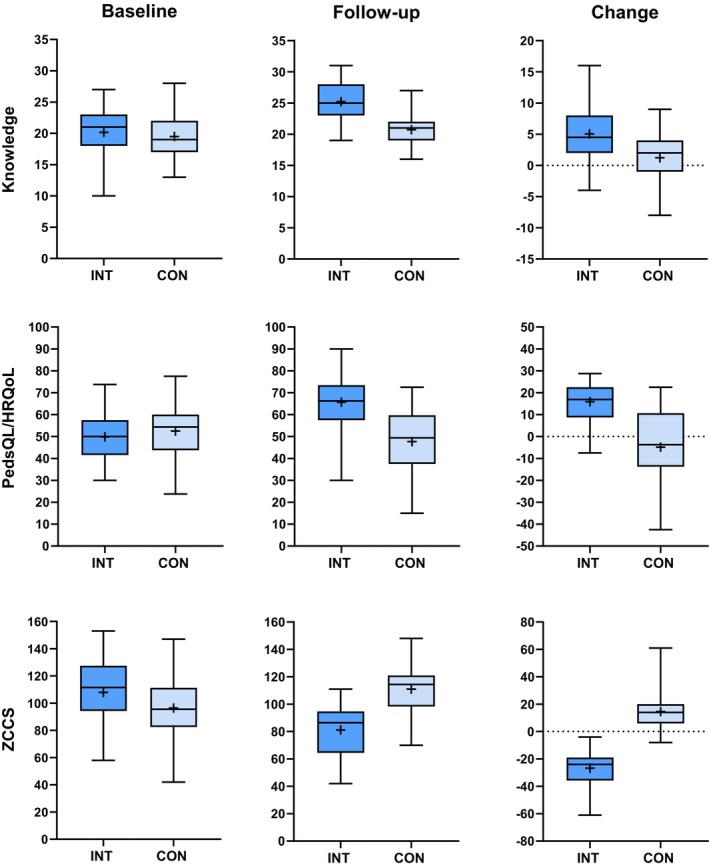
Median, mean, interquartile range, and range of caregiver knowledge (top), Pediatric Quality of Life Inventory (PedsQL) health‐related quality of life (HRQoL) (middle), and total Zimbabwe Caregiver Challenges Scale (ZCCS) (bottom) at baseline (left) and at the follow‐up (middle), with change in score shown on the right; this is the difference between the two assessments for the intervention (INT; dark blue) and control (CON; light blue) groups. The median is shown as a bar in the box and the mean as a cross.

Caregiver knowledge about CP improved significantly, more so in the intervention group than in the control group (Table 2 and Figure 1; *p* < 0.001; Cohen's *d* = 0.98). The knowledge level at the baseline was high, with approximately 20 correct answers out of 36 in both groups, with a mean increase close to five in the intervention group and one in the control group.

**TABLE 2:** Caregiver knowledge score, PedsQL, and ZCCS total and subscale scores for the intervention and control groups at baseline and follow‐up, and the change between groups.

	Intervention group *n* = 48				Control group *n* = 46				Change between groups	
	Baseline	Follow‐up	Change	*p*	Baseline	Follow‐up	Change	*p*	*p*	Cohen's *d*
Caregiver knowledge	20.2 (3.6)	25.2 (2.8)	5.0 (4.2)	< 0.001	19.5 (3.7)	20.7 (2.4)	1.2 (3.6)	0.025	< 0.001	0.98
PedsQL										
Total score (*n* = 36)	53.9 (10.9)	64.3 (10.6)	10.5 (8.7)	< 0.001	54.0 (13.4)	50.7 (15.1)	−3.2 (17.6)	0.225	< 0.001	0.56
Parent HRQoL (20 items)	49.8 (10.6)	65.7 (11.5)	15.9 (8.5)	< 0.001	52.5 (12.5)	47.7 (15.7)	−4.9 (16.1)	0.046	< 0.001	1.15
Family functioning (8 items)	59.2 (23.4)	56.0 (21.5)	−3.3 (24.4)	0.359	52.0 (21.6)	54.5 (20.2)	2.5 (26.9)	0.529	0.139	−0.23
ZCCS										
Total score (*n* = 33)	108.0 (23.3)	81.2 (18.8)	−26.8 (14.6)	< 0.001	96.4 (21.8)	111.0 (18.1)	14.6 (12.5)	< 0.001	< 0.001	−3.2
Physical (5 items) and financial (4 items)	32.4 (9.4)	23.5 (8.2)	−8.9 (4.4)	< 0.001	30.2 (9.5)	32.4 (7.3)	2.2 (6.5)	0.028	< 0.001	−2.0
Family relations (5 items)	17.1 (4.9)	12.46 (4.2)	−4.7 (3.6)	< 0.001	14,3 (5.2)	16.8 (4.8)	2.5 (4.2)	0.001	< 0.001	−1.8
Concerns for the child (14 items)	46.5 (9.7)	36.7 (8.2)	−9.8 (8.2)	< 0.001	43.1 (9.4)	50.0 (8.9)	7.0 (7.9)	0.001	< 0.001	−2.1
Community participation (3 items)	11.9 (3.3)	8.5 (3.2)	−3.3 (2.9)	< 0.001	8.8 (3.0)	11.7 (2.7)	3.0 (2.8)	0.001	< 0.001	−2.2

*Note*: Data are mean (SD). Intragroup differences between assessments were analysed using a paired *t*‐test; between‐group differences were analysed by comparing the change scores across groups using an independent *t*‐test. Abbreviations: HRQoL, health‐related quality of life; PedsQL, Pediatric Quality of Life Inventory; ZCCS, Zimbabwe Caregiver Challenges Scale.

### PedsQL Family Impact Module

The PedsQL Family Impact Module total change score differed significantly between the intervention and control groups (Table 2 and Figure 1; *p* < 0.001; Cohen's *d* = 0.56). The total score increased significantly (mean = 10.5 [SD = 8.7]; *p* < 0.001) in the intervention group, while it tended to decrease in the control group. This was driven by the large between‐group difference in the HRQoL scale (*p* < 0.001; Cohen's *d* = 1.15) and no differences in the family functioning subscale change score between groups.

### Caregiver burden and stress

The total ZCCS score was significantly reduced in the intervention group (mean = −26.8 [SD = 14.6]); *p* < 0.001) while it increased in the control group (mean = 14.6 [SD = 12.5]); *p* < 0.001), resulting in a large change score difference between groups (Table 2 and Figure 1; *p* < 0.001; Cohen's *d* = −3.2). All four subscale scores contributed to the large difference, with a Cohen's *d* between 1.8 and 2.2.

### Caregiver skills

Video assessments were only performed for children who required assistance for feeding or dressing (Manual Ability Classification System levels III–V), with 12 participants from the intervention group and 16 from the control group. Caregiver skills were limited at baseline, with a total around 50% of maximum (max = 15) (Table S1). The total feeding score and acknowledging the child's signals during both dressing and feeding improved significantly in the intervention group but not in the control group. Just considering the child's signals during dressing showed significantly greater improvements across groups.

### Correlations across caregiver outcomes

Correlations between the change in caregiver knowledge and changes in the PedsQL and ZCCS total and subscales are shown in Table 3. With both groups combined, caregiver knowledge correlated strongly with the ZCCS total and subscale scores, particularly the physical and finance subscales. These also correlated with the HRQoL subscale, but not with the total PedsQL score or the family functioning subscale. The latter did not correlate with any other outcome, while there were strong correlations between the HRQoL and ZCCS total scale and subscales.

**TABLE 3:** Pearson's rank correlation coefficients for the change scores of caregiver knowledge and PedsQL and ZCCS, and their respective subscales.

	PedsQL total	HRQoL	Family functioning	ZCCS total	Physical and financial	Family relations	Concerns for child	Community participation
Knowledge	0.20	0.22*	0.09	−0.39**	−0.46**	−0.23*	−0.27**	−0.36**
HRQoL			0.17	−0.52**	−0.44**	−0.45**	−0.43**	−0.44**
Family function		0.17		−0.01	−0.10	0.01	0.01	0.07

Abbreviations: HRQoL, health‐related quality of life; PedsQL, Pediatric Quality of Life Inventory; ZCCS, Zimbabwe Caregiver Challenges Scale.

* *p* < 0.05.

** *p* < 0.001.

### Correlations between caregiver and child outcomes

The age of the child did not correlate with any of the caregiver outcomes, while the GMFCS level correlated with the ZCCS (total) and community participation change scores (*ρ* = −0.43 [*p* = 0.02] and *ρ* = −0.41 [*p* = 0.02] respectively), indicating that more severe functional limitations of the child were associated with greater challenges for the caregiver. Correlations between changes in caregiver outcomes with changes in child outcomes (Table 4),^20^, ^21^ with both groups combined, showed that all three caregiver outcomes, that is, knowledge, HRQoL, and ZCCS, correlated with the 66‐item Gross Motor Function Measure and Pediatric Evaluation of Disability Inventory child and caregiver assistance self‐care and social functioning scores. The ZCCS also correlated with the Pediatric Evaluation of Disability Inventory Caregiver Assistance Scale for mobility and with Picture My Participation attendance. The latter also correlated with HRQoL.

**TABLE 4:** Pearson's correlation coefficients for the change scores of caregiver knowledge, HRQoL, and ZCCS, and the change scores of the child outcomes for the GMFM, PEDI UG (six subscales), and PMP attendance.

	GMFM	PEDI mobility	PEDI self‐care	PEDI social functioning	PEDI CA mobility	PEDI CA self‐care	PEDI CA social functioning	PMP attendance
Knowledge	0.28**	0.08	0.37**	0.35**	0.20	0.36**	0.45*	0.16
HRQoL	0.21*	0.19	0.44**	0.44**	0.19	0.48**	0.33**	0.32**
ZCCS total	−0.27**	−0.10	−0.47**	−0.56**	−0.31**	−0.52**	−0.54**	−0.45**

Abbreviations: GMFM, Gross Motor Function Measure; HRQoL, health‐related quality of life; PEDI CA, Pediatric Evaluation of Disability Inventory Caregiver Assistance; PEDI UG, Pediatric Evaluation of Disability Inventory Ugandan version; PMP, Picture My Participation; ZCCS, Zimbabwe Caregiver Challenges Scale.

* *p* < 0.05;

** *p* < 0.001.

## DISCUSSION

As demonstrated, the Akwenda Intervention Program had a large impact on the HRQoL of caregivers, including perceived physical, emotional, social, and cognitive functioning. HRQoL improved only in the intervention group, leading to large between‐group differences. Comprehensive and multidimensional models of caregiver health have been developed, highlighting the influence of sociodemographic, disability‐related, and psychosocial factors.^1^, ^4^, ^5^ Similar themes have emerged in qualitative studies of mothers caring for children with CP in South Africa,^15^ and in low‐ and middle‐income settings in India.^33^ The caregivers in our cohort were exposed to many of the identified sociodemographic risk factors,^4^ such as low income (83%), being mothers (53%), being divorced or widowed (32%), and having a low level of education (58% primary school or lower). In addition, the disability‐related factors of the child causing high caregiving demands, such as challenging child behaviour, functional limitations, and co‐occurring impairments, have a strong impact on caregiver‐perceived physical, social, and emotional functioning.^1^, ^5^ Nearly half of the children in this study were classified in GMFCS levels III to V at baseline, indicating severe mobility constraints. Consequently, caregivers were exposed to high caregiving demands, which, combined with their vulnerable social situation, further affected their perceived psychosocial functioning. Sociodemographic and disability‐related factors were not directly addressed by the intervention and should have remained relatively stable throughout the study, unless affected by other external factors. One example was the intervention's aim to improve the child's functioning and participation, resulting in improvements in the child's gross motor function, self‐care, and social functioning,^20^ along with improved participation in activities of daily living.^21^ In this study, we observed a correlation between these improvements and caregiver burden of care (ZCCS) and HRQoL. The child's GMFCS level was also related to the ZCCS, indicating that caregiver burden directly reflected the child's degree of functional limitation. However, the direction of causality may also operate in the opposite direction because caregiver functioning in everyday life may also influence the child's outcomes.^12^

Caregiver burden and stress is one of the main factors influencing the health and well‐being of caregivers of children with a disability.^1^, ^4^, ^33^ The intervention significantly reduced the overall caregiver burden and all four ZCCS subscores, while there was an increase in the control group resulting in large between‐group differences (Cohen's *d* = 1.8–3.2). There were strong correlations between the reduction in caregiver burden (ZCCS) and improvements in the total PedsQL Family Impact Module and HRQoL scales. This suggests that the reduction in perceived burden and stress had a positive effect on the perceived psychosocial functioning of caregivers.

The primary focus of our intervention was on the psychosocial factors known to mediate or moderate caregiver health and quality of life.^4^ Social and emotional support have been identified among the strongest predictors of caregiver stress and quality of life.^1^, ^4^, ^5^, ^33^ A key aspect of the Akwenda Intervention Program was to empower caregivers by establishing groups with other caregivers of children with CP. The importance of such support groups is well recognized in community‐based rehabilitation guidelines.^34^ During the 12‐month intervention, caregivers met other caregivers of children with CP, many for the first time, allowing them to share experiences, provide mutual support, and reduce social isolation. Research on self‐help groups for caregivers of children with disabilities in Kenya suggests that such groups are powerful means of empowerment.^17^ In Ghana, the improvement of caregiver HRQoL was also partly achieved through participatory group support providing an alternative social network, which helped caregivers realize that they were ‘not on their own’.^12^, ^14^ Learning about their own child's condition, observing other children, and the group dynamic of sharing experiences with others shifted caregiver attitudes towards their children, promoting acceptance and hope, and alleviating isolation.

The education of caregivers to understand their child's health condition, and training to achieve effective strategies to promote the child's development, were central to the intervention. The group sessions improved the level of knowledge more in the intervention group than in the control group. The strong correlation between improved knowledge and a reduction in caregiver burden and stress, and increase in HRQoL, suggests that caregivers' improved understanding of their child's condition and care needs positively influenced their perceived burden and psychosocial functioning. This finding aligns with the caregiver training programmes conducted in Ghana, which led to better understanding of the child's health condition and improved caregiver HRQoL.^12^, ^14^

The Akwenda Intervention Program specifically targeted the negative attitudes and stigma towards children with disabilities in four caregiver group sessions led by a social worker, and two sessions for community stakeholders. The communication and advocacy for social and behavioural change activities probably shifted caregiver self‐perception, helping them move beyond feelings of guilt and shame. Engaging with other caregivers and being treated with respect by community leaders may have also fostered a sense of community and empowerment.

The positive impact of the intervention on the perceived psychosocial functioning of caregivers in our study aligns with findings from the caregiver training programme in Ghana, which also used the PedsQL Family Impact Module and showed improvements in caregiver HRQoL.^14^ However, there were notable differences between the two studies. In the Ghanaian cohort, baseline PedsQL scores were much lower than in our study (12.5 vs 53.9), improving to 51.4 after the intervention. This disparity might be due to differences in prior knowledge or variations between the two cohorts. For instance, only 13% of Ghanaian caregivers had prior knowledge of CP, while the children of Ugandan caregivers had been diagnosed several years earlier, and caregivers had been informed several years earlier, participating in multiple follow‐up assessments.^18^, ^23^ These differences suggest that caregiver training programmes can have a significant positive impact, whether caregivers are completely unfamiliar with the condition or have some prior knowledge.

### Strengths and limitations

This study is the first reported randomized controlled trial focused on improving the mental, physical, and social functioning of caregivers of children and young people with CP in a rural, resource‐constrained setting in sub‐Saharan Africa. It has several strengths, including strong adherence to methodological guidelines for complex interventions in real‐world settings,^35^ such as a randomized, assessor‐blinded, intention‐to‐treat design, equivalent study arms, low attrition, high compliance, trial preregistration, and outcome measures chosen based on the goals of the intervention.

However, the study setting presented some challenges. Participants were assigned to geographical clusters for the intervention workshops organized in groups of caregivers living near the workshop venue, and to reduce contamination between groups. Although assessors were blinded to the randomized controlled trial design and had no vested interest in the outcomes, they could have unintentionally been influenced by contextual factors, like geography or treatment details such as assistive devices, which might have compromised the blinding process. We used the protocol for reporting randomized trials and not the extended protocol for reporting cluster‐randomized trials, which might have influenced the statistical analysis.^36^

Both the PedsQL Family Impact Module and ZCCS primarily focus on the problems or challenges caregivers face, emphasizing the degree of these negative experiences. However, they fail to capture the positive aspects of caregiving or the potential improvements in these experiences after an intervention. Reducing the negative impact of caregiving does not necessarily equate to enhancing the positive experiences; the absence of ill‐being is not equivalent to high well‐being.^37^ Despite the name, most items of the HRQoL scale of the PedsQL Family Impact Module are more closely associated with biopsychosocial functioning. In retrospect, we recognize that a more balanced perspective, which includes the positive dimensions of the quality of life and well‐being of caregivers, would have been valuable. We recommend that future studies consider the design and selection of instruments that can comprehensively assess both the negative and positive aspects of caregiving.

Another challenge encountered in our study was the absence of validated methods to assess caregiver knowledge and skills. This gap arose from the nature of the intervention and the specific information being conveyed to families, prompting the research team to develop a custom caregiver knowledge questionnaire and a video scoring protocol, which were not validated. Furthermore, the small sample size for the feeding and dressing videos limited the statistical power to detect significant differences.

## CONCLUSIONS

The Akwenda Intervention Program led to reductions in caregiver burden and stress, and improvements in the perceived psychosocial functioning of caregivers of children with CP. Over a 12‐month period, caregivers participated in bimonthly group sessions that improved their knowledge and fostered emotional and social support through peer interactions. The impressive impact of this participatory caregiver programme can serve as a model and inspire similar initiatives aimed at improving the lives of caregivers and children in other low‐resource settings.

## Supporting information


**Appendix S1:** Implementation of the Akwenda Intervention Program for children and young people with cerebral palsy

**Appendix S2:** Caregiver Knowledge & Understanding Questionnaire

**Appendix S3:** Protocol to evaluate caregivers' performance in feeding and dressing

**Figure S1:** Study flow chart.

**Table S1:** Caregiver skill scores for dressing and feeding filmed at baseline and follow‐up

## Data Availability

The data that underlie the results reported in this article are described at the Swedish National Data Service. Data are made available upon request after ensuring compliance with relevant legislation. https://doi.org/10.48723/a8yn‐zs31.
