# Supplementary material for: Impact of the Akwenda Intervention Program for cerebral palsy on caregiver‐perceived burden, stress, and psychosocial functioning: A cluster‐randomized trial in Uganda
Source: Dev Med Child Neurol. 2025 Jun 14;67(9):1206–16. doi: 10.1111/dmcn.16368 (PMC12336398; doi:10.1111/dmcn.16368)
Supplement: Supplementary file 1 — Appendix S1: Implementation of the Akwenda Intervention Program for children and young people with cerebral palsy [file DMCN-67-1206-s005.docx]

**Implementation of the Akwenda intervention program**

**for children and young people with cerebral palsy**

The Akwenda Intervention is a broad and holistic program previously described in detail in the study protocol together with underlying principles and goals.^1^ It comprised five intervention components:

1. Caregiver-led workshops
2. Therapist-led group sessions
3. Provision of technical assistive devices (TADs)
4. Goal directed training
5. Communication and advocacy for behavioural and social change (C&A)

All caregivers started the intervention in the same month and completed the intervention twelve months later. Caregivers were divided into four geographical groups. The venue for each group was a community health centre or visiting point close to the members of the group. Each group attended a weekly session for the first two weeks every month; the first session was a caregiver-led workshop and the second session was a therapist-led practical session with the same theme.

**Caregiver-led workshops and therapist-led practical sessions**

The intervention centred around a series of seven caregiver-led workshops, delivered by caregiver facilitators from the Iganga area who had received 80 hours of training which equipped them to run the workshops independently without a therapist present. These caregiver-led workshops provided information on a variety of topics, each topic building on the previous one (see Table 1). One week after each workshop, caregivers attended a therapist-led practical group session where caregivers practiced what they had learned during the workshop. The caregiver facilitators assisted the therapist during this group session.

During the therapist-led group sessions, the therapist first revised material covered in the caregiver-led workshop and linked this to previous sessions. Following this recap, the therapist carefully demonstrated the practical activity (e.g. preparation and handling techniques for everyday activities; dressing and undressing; feeding; play; communication etc.) on each child whilst the other caregivers and children observed and asked questions about that specific child. In this way, caregivers learnt how CP affected each child’s body in a different way and how the principles, techniques and ideas covered in the workshop could be modified and adapted for each child. Children with similar abilities and clinical presentations were then grouped together into smaller groups where the therapist and caregiver facilitators assisted the caregivers in practicing these skills with their child. The emphasis was on problem-solving in applying the principles taught in the caregiver-led workshops to each child/young person. A “child-active” approach was adopted where each child was encouraged to be actively engaged in the task or activity based on the child’s clinical condition and functional limitations.

| **Caregiver-led workshops followed by Therapist-led workshops**  (same theme) | | |
| --- | --- | --- |
| **#** | **Title of workshop** | **Content of workshop** |
| 1 | What is CP and how does it affect my child? | - Be able to explain what CP is to a family member - Know the GMFCS Level the child falls into - Know what kind (subtype) of CP the child has - Understand the possible causes of CP, and what does not cause CP, especially that CP is not a disease and not her fault |
| 2 | CP as a way of life | - What does “Caring for child with CP is a way of life” mean - How children with CP change over time - How we can do everyday things in a way that is helpful to children with CP - Helpful ways to hold and move children with CP - Helpful ways to put children with CP in different positions |
| 3 | Getting my child’s body ready to move and be active | - How to get a child’s body ready to move and play - How to position a child in a helpful way - How to use these ideas in everyday activities |
| 4 | Eating and drinking: making mealtimes safe and comfortable for my child | - How to best position a child with CP for feeding - Why it is important to feed a child with CP slowly - How to prepare the child’s lips and mouth for eating - How to help a child with CP to eat better with a spoon - How to help a child with CP to learn to chew - How to help a child feed himself - How to help a child with CP learn to drink from a cup - How to help children who drool |
| 5 | Communication: my child and I understanding each other | - What we mean by communication - The different ways we can communicate - How to encourage a child with CP to communicate - How to help a child with CP to understand better - How to help a child with CP to talk better - Helping children to use signs |
| 6 | Play: unlocking my child’s potential | - What is play and why it is important for a child with CP - How children usually play - Why it is difficult for children with CP to play - How we can make it easier for all children with CP to play - What we can play with |
| 7 | CVI: Understanding where and what my child can see | - Understand vision difficulties - What does CVI stand for and what does it mean - How to know if a child has a CVI - How to play with a child with a CVI - How we use what the child sees best in everyday life |

**Table 1: Summary of material covered in the caregiver-led and therapist-led workshops**

Therapist-led workshops on goal setting; developing action plans to achieve the goals; the provision of TADs; and training in the use and maintenance thereof together with workshops on child health, nutrition and epilepsy were interspersed in between the parent-led workshops and therapist-led group practical sessions as shown in Table 2.

The caregiver-facilitators were the core of the intervention. They were themselves primary caregivers of children with CP. Caregivers know their children best and the intention of the carer-to carer aspect of the Akwenda intervention was to build on this lived experience and not only empower caregivers with knowledge and skills on how to best care for their children, but through peer-support, to decrease caregivers’ isolation and improve their mental health.

**Table 2: Summary of material covered in the special therapist-led workshops**

| **Special Therapist-led workshops** (In addition to those in Table 1) | | |
| --- | --- | --- |
| 8 | Technical assistive devices session 1 | - Understand what the purpose of TADs is - Understand why not all children will receive TADs - Know the basics of setting up the device, cleaning, and maintenance - Be able to demonstrate how to place the child in the device |
| 9 | Technical assistive devices session 2 | - Caregiver able to fit a child in a variety of assistive devices - Caregiver able to integrate information and skills learnt in earlier workshops in applying and using TADs - Understand when and how to use a TAD, and for how long |
| 10 | Goal setting session 1 | - Understand why setting goals is helpful and important - Understand what a goal looks like - Be able to set three (or 4 if they have TADs) goals |
| 11 | Goal setting session 2 | - Be able to list the goals they set for their child - Explain the action plan of what will be practiced at home to achieve the goals |
| 12 | Healthy diet and healthy eating  &  Understanding epilepsy | - Know the seven major classes of nutrients - Know how to prepare a meal from locally available nutrient dense foods - Understand responsive feeding practices (e.g. frequency of feeding, creating a safe and clean environment for feeding) - How to recognise a seizure - Understand misconceptions related to epilepsy and how to deal with stigma - Know when and where to seek care for a child with seizures - Understand the role of traditional healers and traditional treatments - Understand anti-epileptic medicine – how to administer; side effects and the importance of adherence |
| 13 | Goal setting session 3 | - Revise action plans - Feedback from caregivers on home practice |
| 14 | Recap | - Summary of what was covered during the year - Be reminded of what they have learnt and taken away from the practical session |

**Provision of technical assistive devices (TADs)**

The therapists together with the caregivers and children assessed the needs for TADs during the baseline assessments. TADs were issued during the seventh month of the intervention immediately after two therapist-led training workshops explaining the use, maintenance, application, and care of assistive devices (see Table 2). No workshops took place in the seventh month as the therapists spent the month visiting all the children in their homes to issue, set up and fit the TADs.

TADs delivered included wheelchairs, standing frames, anterior and posterior walking frames, benches, postural support chairs, toilet seats, parallel bars, knee and hand pads, ankle foot orthoses, hand and elbow splints, soft splints for upper and lower limbs, special beds, and mattresses, cut out cups, and spoons. Most devices were made by local artisans from either wood or using appropriate paper technology. Prior to fitting of standing frames and hand and foot orthoses, therapists applied serial casting to increase soft tissue length for all children who had soft tissue tightness. Casting for hand and foot orthotics was done after the serial casting. Ramps were built in the homes of all children using wheelchairs or walking devices. The project supplied materials and local builders built the ramps together with the families.

Delivering of the TADs was originally planned to occur at the 5^th^ month, but was delayed because of logistical issues in procuring and manufacturing the various items. The flow of the programme were adapted accordingly by moving up sessions one month until the TADs were ready. This change in order did not disturb the implementation of the program.

**Goal directed training**

Three therapist-led workshops on goal setting were parts of the intervention (see Table 2). In the first workshop, the concept and value of goals was explained to parents and parents had to set three goals for their child, and a fourth goal if the child had been issued with a TAD. In the second goal setting workshop goals were refined into GAS goals and in the following month, parents had to work out an action plan for achieving the goals. These two workshops proved to be the longest and hardest workshop for the therapists, taking an average of seven hours to complete, as each parent in the group required individualised assistance in working out GAS goals and action plan. The time to achieve a goal was set for four months which corresponded to the follow-up assessment of the intervention. A third goal setting workshop took place a month after the GAS goals were set and action plans drawn up. The focus of this third workshop on goal setting was a “check in” with caregivers to review the action plans and practice in the home setting. Originally, the goal setting sessions were planned earlier in the programme, but they were delayed to include goals on the use of TADs.

**Communication and Advocacy for behavioural and social change (C&A)**

**Caregiver C&A sessions**

Caregivers of the children with CP in the intervention group participated in four C&A meetings, each lasting 90 minutes and led by the community social worker. These took place in months one, four, five, and six on the same day as one of the caregiver-led workshops. This meant that on these specific days, caregivers spent the whole day at the venue.

Topics for the C&A sessions were:

1. rights of children with disabilities with a focus on human rights and abuse of children with CP in the community and in the home
2. mapping available health, education, and legal services in the community
3. identifying barriers to accessing services for children with disabilities
4. developing an action plan to improve services for children with disabilities in their community

A structured programme with objectives and key take-away points for each C&A workshop was drawn up by the community social worker assisted by one of the authors (GS). Typically, the workshop comprised an icebreaker, an information session, a participatory activity followed by a discussion and summary. The community social worker who led the workshops was particularly skilled in facilitating discussion and promoting participation and engagement of all workshop participants.

The focus of the first two workshops was on awareness raising and information regarding the rights of the children with disabilities and mapping out current services in their communities. The focus of the final three workshops was on developing solutions and action plans for improving access to services for their children. Since caregivers remained in their four geographically based groups, they identified problems particular to their communities with specific action plans.

**Community stakeholder C&A sessions**

Thirty-eight community representatives, including political, administrative, cultural, and religious leaders, educators, health workers, police, disability NGO/CBO representatives, district and sub-county technical personnel participated in two C&A meetings led by the community social worker. The first session took place in the second month of the intervention and focused on the rights of children with disabilities and the stigma they meet leading to discrimination and barriers limiting their participation. It was led by the community social worker assisted by the caregiver facilitators who delivered a presentation on CP.

The second meeting took place during the ninth month. This was a joint dialogue session with the community stakeholders and representatives of each of the four caregiver groups. The caregivers discussed their action plans with the community stakeholders.

To sensitize the community to disability issues, information on cerebral palsy was delivered through a one-hour talk show on six community radio stations by the community social worker and one of the authors (AKM) at the start of the intervention. Messages included information how the Akwenda Intervention intended to assist children with cerebral palsy in Iganga-Mayuge. A telephone number of the community social worker was shared with the listeners at the end of each radio talk show.

**Time frame**

The intervention ran for 12 consecutive months, starting in October 2021, after base line assessments, and finishing in September 2022, followed by outcome assessments.

**Human resources**

A significant amount of time was spent on training and coaching the Caregiver Facilitators and the therapists who conducted the intervention. Covid-19 pandemic delayed the start of the intervention by 16 months, necessitating refresher training for both the Caregiver Facilitators and the therapists.

Five caregivers were trained according to the Carer-2-Carer concepts previoulsy developed in South Africa.^2^ The training occurred at two instances 40 and 80 hours, respectively. It was led by a Master Trainer (also a parent) from South Africa who travelled to Iganga for two periods. One of the caregiver facilitators dropped out due to health reasons. The caregiver facilitators worked in pairs, with one pair assigned to each therapist during the therapist-led sessions.

Four therapists were selected from an initial seven day practical Introductory Course on Cerebral Palsy in 2019. In addition, they received a two week training course focused on equipping and preparing them to run the intervention. A detailed training manual was compiled for the therapists. All the training took place in Iganga and was conducted by one of the authors (GS), a physiotherapist based in South Africa. A nine day refresher training for the therapists and the caregiver facilitators was conducted via Zoom in 2021. One of the four therapists dropped out and went on maternity leave.

Two therapists were responsible for the intervention, and each had two groups they were responsible for each month. In the four months when there was not a caregiver-led workshop, each therapist ran four sessions per month. The therapists rotated each month. In this way, both therapists worked with all four groups.

The third therapist was responsible for coaching and mentoring the Caregiver Facilitators throughout the intervention. He met the Caregiver Facilitators for two days each month to revise the material and plan presentations before they ran their workshops. He was not present during the workshop but held a debriefing session with them after they had run their workshops each month.

All three therapists attended two online coaching and mentoring sessions a month with the trainer (GS). The purpose of these sessions was to revise the content and the structure of the monthly session and to debrief after each group session. The training manual for the intervention was updated each month based on lessons learnt from each group session.

One of the authors (GS) had online coaching sessions with the social worker to prepare for each of the C&A workshops. These sessions also included a reflection and debriefing session in preparation for the following C&A session.

The use of caregiver facilitators and working with children in groups decreased the number of therapist hours required to run the intervention. A total of 150 therapist hours were required to run the whole program. In addition, a the third therapist not involved in the practical sessions monthly supported and mentored the parent facilitators.

**Compliance**

Caregiver compliance was 93% for the seven caregiver-led workshops, and 95 % for the fourteen therapist-led workshops. Nineteen caregivers attended all twenty-one workshops, and nineteen missed only one workshop. Caregiver compliance to the four C&A workshops led by the community social worker was 93%. Forty stakeholders were invited to both C&A sessions, and 38 of these participated in each session, giving a compliance of 95%.

**Reimbursement**

Transport was a significant economic barrier for caregivers to come with or without their child to the venue where the intervention sessions were held although efforts had been made to cluster families close to the venue. To support participation caregivers were given transport reimbursement of 15,000 Uganda shillings (4 USD). They were also provided with breakfast when participatin in morning workshops, and an additional lunch on the days they attended the C&A workshop in the afternoon.

Stake holders were provided with 50,000 Uganda shillings (13 USD) for transport reimbursement since the venue often was distant from their working offices. They were also served break tea and lunch.

References

1. Saloojee G, Ekwan F, Andrews C, Damiano DL, Kakooza-Mwesige A, Forssberg H. Akwenda intervention programme for children and youth with cerebral palsy in a low-resource setting in sub-Saharan Africa: protocol for a quasi-randomised controlled study. 2021. 0:e047634.doi:10.1136/bmjopen-2020-047634.

2. Saloojee G, Bezuidenhout M. Parent-led services and community-based peer supporters for children and adults with disabilities: experiences from two programmes. In: Padarath A, Kathard H, Lorenzo T, et al., eds. South African Health Review 2020. Durban: Health Systems Trust 2020.
