# Supplementary material for: Impact of the Akwenda Intervention Program for cerebral palsy on caregiver‐perceived burden, stress, and psychosocial functioning: A cluster‐randomized trial in Uganda
Source: Dev Med Child Neurol. 2025 Jun 14;67(9):1206–16. doi: 10.1111/dmcn.16368 (PMC12336398; doi:10.1111/dmcn.16368)
Supplement: Supplementary file 2 — Appendix S2: Caregiver Knowledge & Understanding Questionnaire [file DMCN-67-1206-s001.docx]

| **CAREGIVER KNOWLEDGE & UNDERSTANDING QUESTIONNAIRE** | | | | |
| --- | --- | --- | --- | --- |
| S/N | STATEMENT | RESPONSE | | |
| 1 | My child has CP because I’m being punished for doing something very bad. | agree | disagree | not sure |
| 2 | Some babies are born with CP because they have had a curse put on them. | agree | disagree | not sure |
| 3 | CP is a disease. | agree | disagree | not sure |
| 4 | It is possible to catch CP from another child who has CP | agree | disagree | not sure |
| 5 | CP can sometimes be inherited from one or both of the child’s parents. | agree | disagree | not sure |
| 6 | A very long labour may cause CP. | agree | disagree | not sure |
| 7 | CP can be caused if you cheat on your partner when you are pregnant. | agree | disagree | not sure |
| 8 | If you have sex when you’re pregnant, you have a higher risk of having a baby with CP. | agree | disagree | not sure |
| 9 | CP can be caused by a lack of oxygen to the brain when the baby is being born. | agree | disagree | not sure |
| 10 | CP can be caused if a mother’s eggs are damaged. | agree | disagree | not sure |
| 11 | Babies who are born very early have more chance of developing CP than babies born at 9 months. | agree | disagree | not sure |
| 12 | CP can be cured. | agree | disagree | not sure |
| 13 | Some of the bigger hospitals have machines which can cure CP. | agree | disagree | not sure |
| 14 | A child will eventually die from having CP. | agree | disagree | not sure |
| 15 | The brain damage from CP stays the same the child’s whole life, however their body can change. | agree | disagree | not sure |
| 16 | CP can be cured with the help from a traditional healer. | agree | disagree | not sure |
| 17 | There are special medicines which can cure CP. | agree | disagree | not sure |
| 18 | All children with CP should go to special school. | agree | disagree | not sure |
| 19 | Going to school won’t help a child with CP who can’t speak. | agree | disagree | not sure |
| 20 | Children in wheelchairs can go to school only if they are toilet-trained. | agree | disagree | not sure |
| 21 | All children with CP should go to school | agree | disagree | not sure |
| 22 | Children with CP must do physio exercises twice every day. This helps them the most. | agree | disagree | not sure |
| 23 | Doing everyday things with my child in helpful ways, like dressing, bathing and feeding, helps my child the most. | agree | disagree | not sure |
| 24 | It is good if my child cries when I am doing exercises because then I know that the exercises are working. | agree | disagree | not sure |
| 25 | The best position for my child to sit or lie in, is the one they are most comfortable in. | agree | disagree | not sure |
| 26 | It is good for my child to practice doing tasks by himself every day, even if he makes mistakes, takes a long time and I have to help. | agree | disagree | not sure |
| 27 | It is only important for children with CP to be in a good position when they are lying down to sleep or rest. | agree | disagree | not sure |
| 28 | It is important for children with CP to always be in a good position because then it will be easier for them to see, communicate and to use their hands. | agree | disagree | not sure |
| 29 | Practicing standing is important for children who can’t stand by themselves because they will eventually learn to walk. | agree | disagree | not sure |
| 30 | Practicing standing is important for all children with CP as it can help to keep their legs strong and straight. | agree | disagree | not sure |
| 31 | If a child with CP has not learned to chew by the time he/she is 5 years old, they will never learn to chew. | agree | disagree | not sure |
| 32 | The only way we can communicate with another person is by speaking in the same language. | agree | disagree | not sure |
| 33 | You don’t only have to use words to communicate with someone. | agree | disagree | not sure |
| 34 | Playing is helpful because children can learn from playing. | agree | disagree | not sure |
| 35 | A child can only play if they can use their hands. | agree | disagree | not sure |
| 36 | Playing with children with CP is not important because they do not know how to play. | agree | disagree | not sure |
