# Supplementary material for: Impact of the Akwenda Intervention Program for cerebral palsy on caregiver‐perceived burden, stress, and psychosocial functioning: A cluster‐randomized trial in Uganda
Source: Dev Med Child Neurol. 2025 Jun 14;67(9):1206–16. doi: 10.1111/dmcn.16368 (PMC12336398; doi:10.1111/dmcn.16368)
Supplement: Supplementary file 3 — Appendix S3: Protocol to evaluate caregivers' performance in feeding and dressing [file DMCN-67-1206-s004.docx]

**Supplement 3:**

**Protocol to evaluate caregivers’ performance in feeding and dressing**

**Background and purpose:**Improving caregiving practices in everyday routines and interactions with children was a key focus of the Akwenda Intervention Program (see supplement 1). This was especially critical for children with limited self-care abilities, i.e., those at MACS levels III – V. To measure the changes resulting from the intervention, a video-based protocol was developed to evaluate caregivers' performance during feeding and dressing activities in the home setting. Videorecording allows for precise observation of behaviour duration, intensity, and patterns. Unlike direct observation, videorecording enables multiple observers to review the data, enhancing objectivity and accuracy in the assessment (Latvala et al 2000).

**Video filming:**Caregivers were filmed while engaging in activities like dressing/undressing or feeding their child. During feeding, they were given a small packet of biscuits and a drink. Each activity was filmed for three minutes. The videos were recorded on tablets or smartphones by the interviewer and uploaded to a secure database. Only caregivers with videos from both baseline and follow-up assessments were included in the analysis.

**Scoring of video films:**The videos were later assessed by two blinded raters who were unaware of whether the child was in the intervention or control group or whether the video was from the baseline or follow-up phase. A coding scheme, developed by one of the authors (GS), was used to assess five observable behaviours:

- Child Positioning: Child is sitting upright with appropriate support
- Caregiver Positioning: Caregiver is comfortable and at the child’s level
- Facilitating Active Participation: Caregiver is engaging child in the activity
- Talking: Caregiver talks to child and explains what is happening
- Acknowledgment: Caregiver pays attention and recognizes child’s signals

Each of these behaviours was scored using a 4-point Likert scale:

- 0 = never
- 1 = sometimes (1%-29% of the time)
- 2 = most times (30%-89% of the time)
- 3 = always (>90% of the time)

The total score ranged from 0 to 15. The assessors underwent training to ensure high interrater reliability (>0.80) by reviewing and discussing scores on 10 practice videos. Following this, they independently coded the study videos. Any discrepancies were resolved by discussion, with a third trained assessor available to mediate as necessary.

Latvala, E., P. Vuokila-Oikkonen and S. Janhonen. "Videotaped recording as a method of participant observation in psychiatric nursing research." J Adv Nurs 31 (2000): 1252-7. 10.1046/j.1365-2648.2000.01383.x. https://www.ncbi.nlm.nih.gov/pubmed/10840260.
