# Supplementary material for: Impact of the Akwenda Intervention Program for cerebral palsy on caregiver‐perceived burden, stress, and psychosocial functioning: A cluster‐randomized trial in Uganda
Source: Dev Med Child Neurol. 2025 Jun 14;67(9):1206–16. doi: 10.1111/dmcn.16368 (PMC12336398; doi:10.1111/dmcn.16368)
Supplement: Supplementary file 4 — Figure S1: Study flow chart. [file DMCN-67-1206-s003.pptx]

## Slide 1
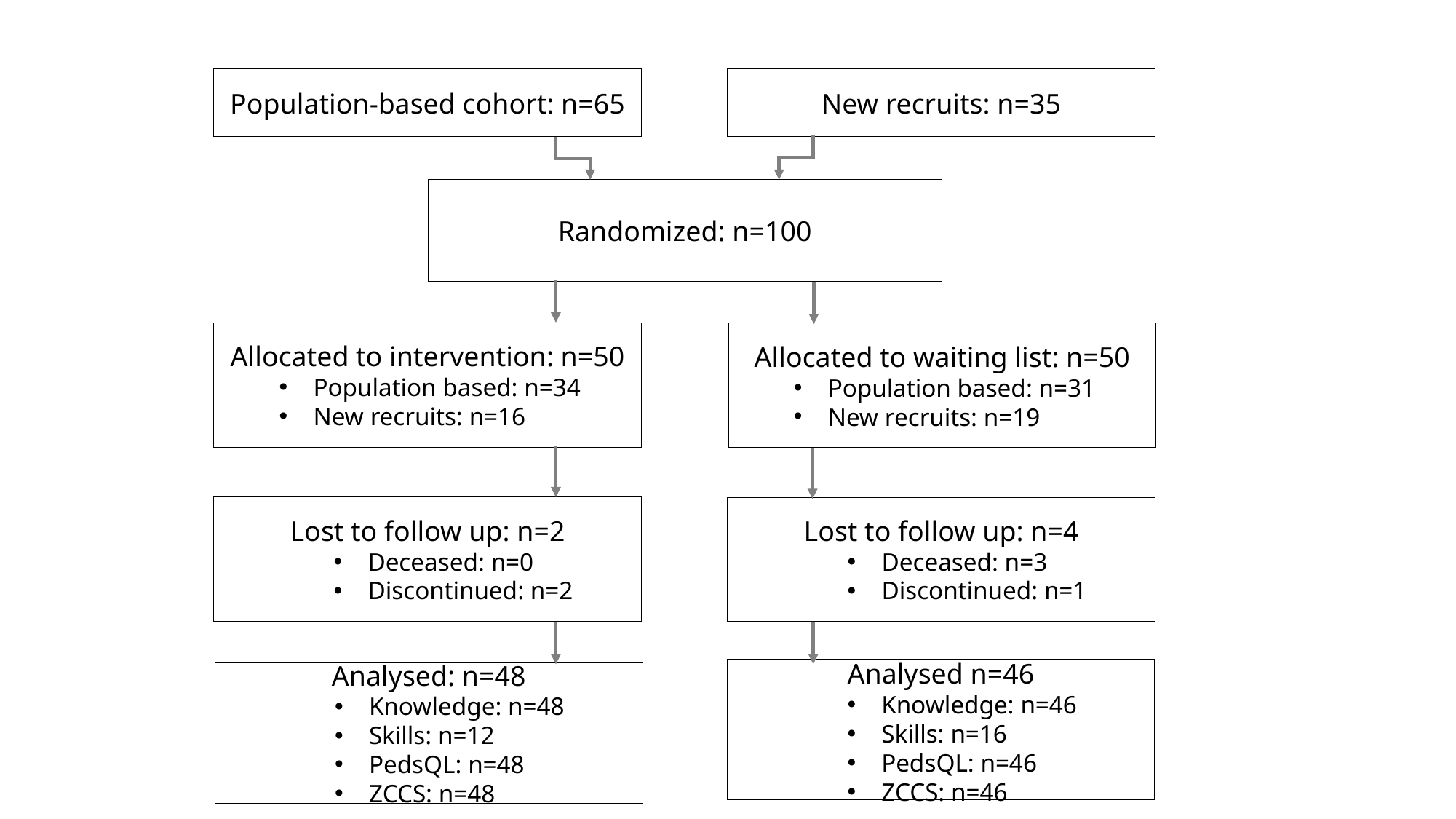

New recruits: n=35
Population-based cohort: n=65
Randomized: n=100
Allocated to intervention: n=50
Population based: n=34
New recruits: n=16
Allocated to waiting list: n=50
Population based: n=31
New recruits: n=19
Lost to follow up: n=4
Deceased: n=3
Discontinued: n=1
Lost to follow up: n=2
Deceased: n=0
Discontinued: n=2
Analysed n=46
Knowledge: n=46
Skills: n=16
PedsQL: n=46
ZCCS: n=46
Analysed: n=48
Knowledge: n=48
Skills: n=12
PedsQL: n=48
ZCCS: n=48
