# Supplementary material for: Impact of the Akwenda Intervention Program for cerebral palsy on caregiver‐perceived burden, stress, and psychosocial functioning: A cluster‐randomized trial in Uganda
Source: Dev Med Child Neurol. 2025 Jun 14;67(9):1206–16. doi: 10.1111/dmcn.16368 (PMC12336398; doi:10.1111/dmcn.16368)
Supplement: Supplementary file 5 — Table S1: Caregiver skill scores for dressing and feeding filmed at baseline and follow‐up [file DMCN-67-1206-s002.docx]

**Supplemental Table 1**: Caregiver skill scores for dressing and feeding filmed at baseline and follow-up.

|  | **INTN** | **INT**  **Baseline** | **INT**  **Follow-up** | **INT**  **Change** | **p** | **CON**  **N** | **CON**  **Baseline** | **CON**  **Follow-up** | **CON Change** | **p** | **t-value** | **p** |
| --- | --- | --- | --- | --- | --- | --- | --- | --- | --- | --- | --- | --- |
| **Video Scoring - Dressing** | | | | | | | | | | | | |
| Total Score | 11 | 6.2 (1.8) | 8.8 (3.5) | 2.6 (4.0) | 0.06 | 16 | 5.6 (3.4) | 5.6 (3.0) | 0.0 (3.1) | 1.00 | 1.91 | 0.07 |
| Child position | 11 | 2.1 (0.8) | 2.3 (0.8) | 0.2 (1.3) | 0.64 | 16 | 2.0 (1.1) | 1.9 (1.2) | -0.1 (1.1) | 0.83 | 0.53 | 0.60 |
| Caregiver position | 11 | 3.0 (0.0) | 2.8 (0.6) | -0.2 (0.6) | 0.34 | 16 | 2.4 (1.2) | 2.2 (1.2) | -0.3 (1.9) | 0.60 | 0.12 | 0.91 |
| Active participation | 11 | 0.2 (0.4) | 0.7 (1.2) | 0.6 (1.4) | 0.22 | 16 | 0.4 (0.9) | 0.6 (1.0) | 0.2 (0.8) | 0.33 | 0.88 | 0.39 |
| Talking | 11 | 0.4 (0.5) | 1.2 (1.2) | 0.8 (1.3) | 0.06 | 16 | 0.3 (0.8) | 0.4 (0.7) | 0.1 (0.6) | 0.43 | 1.90 | 0.07 |
| Acknowledging signals | 11 | 0.6 (0.8) | 1.8 (1.1) | **1.3 (1.3)** | **0.01** | 16 | 0.6 (1.0) | 0.6 (0.9) | 0.0 (0.8) | 1.00 | 3.06 | **0.01** |
| **Video Scoring - Feeding** | | | | | | | | | | | | |
| Total Score | 9 | 6.7 (3.0) | 9.7 (2.7) | **3.0 (3.2)** | **0.02** | 10 | 7.3 (1.6) | 8.1 (2.3) | 0.8 (2.5) | 0.34 | 1.68 | 0.11 |
| Child position | 9 | 1.8 (1.1) | 2.2 (0.7) | 0.4 (1.2) | 0.31 | 10 | 2.5 (0.7) | 2.0 (0.8) | -0.5 (0.7) | 0.05 | 2.07 | 0.05 |
| Caregiver position | 9 | 3.0 (0.0) | 3.0 (0.0) | 0.0 (0.0) | 1.00 | 10 | 2.9 (0.3) | 2.9 (0.3) | 0.0 (0.5) | 1.00 | 0.00 | 1.00 |
| Active participation | 9 | 0.6 (1.1) | 1.3 (1.0) | 0.8 (1.7) | 0.21 | 10 | 0.7 (0.7) | 1.2 (0.9) | 0.5 (1.2) | 0.21 | 0.42 | 0.68 |
| Talking | 9 | 0.3 (0.5) | 1.0 (1.0) | 0.7 (1.0) | 0.08 | 10 | 0.3 (0.5) | 0.6 (0.7) | 0.3 (0.8) | 0.28 | 0.88 | 0.39 |
| Acknowledging signals | 9 | 1.0 (0.9) | 2.1 (0.8) | **1.1 (0.6)** | **<0.001** | 10 | 0.9 (0.7) | 1.4 (1.0) | 0.5 (1.3) | 0.24 | 1.30 | 0.21 |

Legend Supplemental Table 1: Data is Mean and ± Standard Deviation. Intra group differences between assessments were analysed by paired t-tests and between group differences were analysed by comparing change scores across groups using independent t-tests.
